# Identification of Social Engagement Indicators Associated With Autism Spectrum Disorder Using a Game-Based Mobile App: Comparative Study of Gaze Fixation and Visual Scanning Methods

**DOI:** 10.2196/31830

**Published:** 2022-02-15

**Authors:** Maya Varma, Peter Washington, Brianna Chrisman, Aaron Kline, Emilie Leblanc, Kelley Paskov, Nate Stockham, Jae-Yoon Jung, Min Woo Sun, Dennis P Wall

**Affiliations:** 1 Department of Computer Science Stanford University Stanford, CA United States; 2 Department of Bioengineering Stanford University Stanford, CA United States; 3 Department of Pediatrics and Biomedical Data Science Stanford University Stanford, CA United States; 4 Department of Biomedical Data Science Stanford University Stanford, CA United States; 5 Department of Neuroscience Stanford University Stanford, CA United States

**Keywords:** mobile health, autism spectrum disorder, social phenotyping, computer vision, gaze, mobile diagnostics, pattern recognition, autism, diagnostic, pattern, engagement, gaming, app, insight, vision, video

## Abstract

**Background:**

Autism spectrum disorder (ASD) is a widespread neurodevelopmental condition with a range of potential causes and symptoms. Standard diagnostic mechanisms for ASD, which involve lengthy parent questionnaires and clinical observation, often result in long waiting times for results. Recent advances in computer vision and mobile technology hold potential for speeding up the diagnostic process by enabling computational analysis of behavioral and social impairments from home videos. Such techniques can improve objectivity and contribute quantitatively to the diagnostic process.

**Objective:**

In this work, we evaluate whether home videos collected from a game-based mobile app can be used to provide diagnostic insights into ASD. To the best of our knowledge, this is the first study attempting to identify potential social indicators of ASD from mobile phone videos without the use of eye-tracking hardware, manual annotations, and structured scenarios or clinical environments.

**Methods:**

Here, we used a mobile health app to collect over 11 hours of video footage depicting 95 children engaged in gameplay in a natural home environment. We used automated data set annotations to analyze two social indicators that have previously been shown to differ between children with ASD and their neurotypical (NT) peers: (1) gaze fixation patterns, which represent regions of an individual’s visual focus and (2) visual scanning methods, which refer to the ways in which individuals scan their surrounding environment. We compared the gaze fixation and visual scanning methods used by children during a 90-second gameplay video to identify statistically significant differences between the 2 cohorts; we then trained a long short-term memory (LSTM) neural network to determine if gaze indicators could be predictive of ASD.

**Results:**

Our results show that gaze fixation patterns differ between the 2 cohorts; specifically, we could identify 1 statistically significant region of fixation (*P*<.001). In addition, we also demonstrate that there are unique visual scanning patterns that exist for individuals with ASD when compared to NT children (*P*<.001). A deep learning model trained on coarse gaze fixation annotations demonstrates mild predictive power in identifying ASD.

**Conclusions:**

Ultimately, our study demonstrates that heterogeneous video data sets collected from mobile devices hold potential for quantifying visual patterns and providing insights into ASD. We show the importance of automated labeling techniques in generating large-scale data sets while simultaneously preserving the privacy of participants, and we demonstrate that specific social engagement indicators associated with ASD can be identified and characterized using such data.

## Introduction

### Background

Autism spectrum disorder (ASD) is a neurodevelopmental disorder characterized by social impairments, communication difficulties, and restricted and repetitive patterns of behavior. Currently, 1 in 44 children in the United States have been diagnosed with ASD, with males 4 times more likely to be affected than females [1,2]. ASD usually manifests in infants and children and presents a wide range of symptoms that vary in intensity from person to person. The heterogeneity of ASD presents a major diagnostic challenge, with clinicians typically employing a combination of lengthy parent questionnaires and clinical observation to evaluate children.

Standard diagnostic mechanisms for ASD are often accompanied by a range of issues that result in long waiting times for results [3-5]. However, in recent years, significant strides have been made in the fields of computer vision and mobile technology, giving rise to the possibility of using home videos of a child's natural behaviors to identify characteristics linked with ASD and enable a more accurate and timely diagnosis [6].

**Figure 1:**
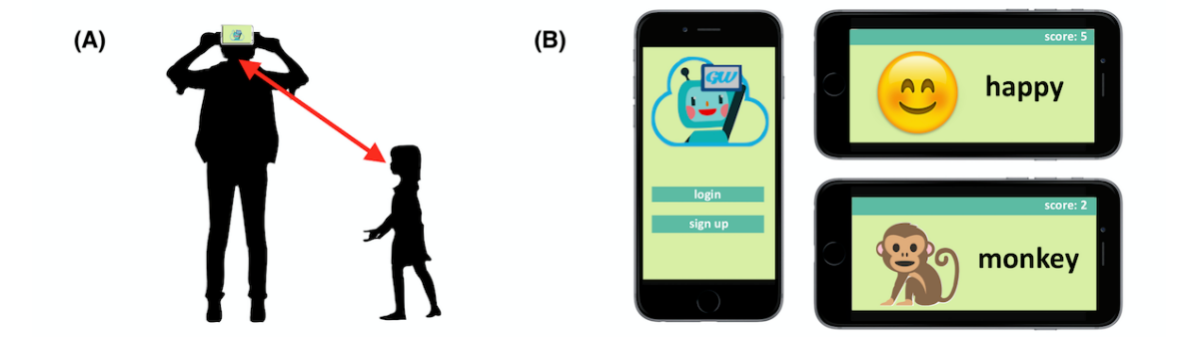
GuessWhat Mobile app. (A) The parent places the mobile phone in a fixed location, allowing the recording of a semistructured gameplay video. (B) The children are presented with a variety of charades prompts, such as emotions and animals.

We previously created a mobile app called GuessWhat, which yields video data of children engaged in socially motivated gameplay with parents in a natural home environment [7-13]. The app presents a charades game, encouraging kids to act out a series of given prompts, such as emotions, sports, or chores. During a game, parents will open the GuessWhat app and place the smartphone on their foreheads, with the front-facing camera pointing at the child; the child then proceeds to act out the prompt displayed on the device while the parent attempts to predict the answer, as shown in Figure 1. The game ends when the 90-second time limit is exceeded. At this point, the parent can view the video recording of the child and is then given the option to share this data with our research team.

The data collection pipeline employed by GuessWhat provides several benefits that make the obtained information amenable to computational analysis. First, although children are performing varied tasks in diverse environments, GuessWhat videos encourage inherent structure, with factors such as the position of the phone camera, location of the child relative to the camera, and game-based social interactions between the parent and child remaining generally consistent between videos. In addition, as children are in a home environment and are unencumbered by bulky hardware such as eye trackers or head mounts, they can interact with their parents and surroundings in a natural manner. As a result, we hypothesize that computer vision algorithms can be designed to monitor socially motivated facial engagement in children during gameplay, allowing effective identification of behaviors, eye contact events, and social interactions potentially correlated with the ASD phenotype.

In this work, we used computational techniques to analyze these videos and identify differences in social interactions between children with ASD and neurotypical (NT) children. We specifically analyzed 2 common social engagement signals that are included in standard clinical diagnostic instruments and can be identified through computer vision methodologies: (1) gaze fixation patterns, which represent the regions of an individual’s visual focus and (2) visual scanning methods, which refer to the ways in which individuals scan their surrounding environment. We performed these tasks without sharing participant videos or private patient information with human annotators.

Ultimately, the development of this system can help improve diagnosis of ASD through automated detection of impaired social interactions, mitigating the problems associated with limited diagnostic resources for neurodevelopmental disorders, especially in regions where access to care is limited [14]. This work also demonstrates the usefulness of game-based approaches and automated labeling methods in preserving privacy, generating large diagnostic data sets, and improving human understanding of complex conditions.

### Prior Work

Researchers have demonstrated the usefulness of video data in providing diagnostic insights into gaze and engagement behaviors associated with ASD. Prior work can generally be divided into three categories: (1) manual annotation methods, (2) eye-tracking systems, and (3) use of structured environments.

#### Manual Annotation Methods

Some studies have used human annotators to label social interaction and engagement information in video frames. Several prior works, such as those by Tariq et al and Leblanc et al, performed manual annotation of behavioral features in home videos, which enabled the creation of classifiers that could identify ASD with high accuracy [15-20]. Chorianopoulou et al collected structured home videos from participants and had expert annotators label the data set with the actions, emotions, gaze fixations, utterances, and overall level of engagement in each video; this information was then used to train a classifier to identify specific engagement features that could be correlated with ASD [21]. Rudovic et al trained a large and generalizable neural network to estimate engagement in children with ASD from different cultural backgrounds [22]. Engagement labels were manually annotated by trained individuals. Although these methods enable the creation of human-vetted, accurate data sets, such approaches require large numbers of trained annotators when implemented on a large scale, which is expensive and time-consuming. In addition, these techniques may compromise the privacy of participants by providing annotators with access to video footage, although some methods have been developed to address privacy concerns with crowdsourced annotations [23,24].

#### Eye-Tracking Systems

Several studies have used eye trackers to identify patterns in gaze and engagement behaviors that may be indicative of ASD or other developmental conditions [25-28]. Pusiol et al showed that deep learning models trained on data collected from a head-mounted eye tracker and camera could be used to classify idiopathic developmental disorder and fragile X syndrome with high precision [29]. Similarly, Riby et al used eye trackers to show that individuals with ASD had atypical gaze patterns when watching movies and cartoons [30]. To counteract artificial movements often associated with facial eye trackers, Noris et al developed a nonintrusive eye-tracking device mounted on a hat that recorded a child's interactions with an interviewer; the study concluded that children with ASD were more inclined to look downward during social interaction than their NT peers [31]. Despite the accuracy and quality of gaze data collected from such systems, eye trackers require custom hardware that can often be expensive and inaccessible, especially for individuals living in resource-limited regions. As a result, these approaches are unlikely to be accessible to the general population.

#### Use of Structured Environments

Hashemi et al explored the use of computer vision algorithms to identify behaviors associated with ASD [32]. A trained clinician administered a series of predefined, structured tasks involving toys and other visual stimuli, while a video camera captured footage of the child's response. A computer vision system that analyzed the child's body orientation and facial movement was able to evaluate the child's engagement with high accuracy. Similarly, Chang et al used the front-facing camera of a mobile device to capture gaze scanning patterns as children watched strategically designed short movies [33]. Automated computer vision techniques were then used to identify differences in gaze patterns between children with ASD and NT individuals. Egger et al also used mobile phones to collect videos of ASD and NT children engaging with short movies. Visual stimuli in movies were carefully designed based on neuroscience principles, and children’s emotional and behavioral responses were computationally analyzed [34]. These works demonstrate effective methods for analyzing engagement patterns without the use of manual annotations or external eye-tracking hardware; however, these studies were conducted with highly structured tasks (eg, carefully selected movies and toys) and controlled environmental factors (eg, Hashemi et al and Chang et al controlled the room lighting and distance of the camera from the participant's face). As a result, the ability of these techniques to translate to natural nonclinical environments and unstructured tasks remains to be explored. In addition, these works do not evaluate engagement and behaviors in social situations.

### Our Contributions

To the best of our knowledge, this is the first study that attempts to obtain diagnostic insights into ASD from social gameplay videos without the use of eye-tracking hardware, manual frame-level annotations, and structured scenarios or environments. We show that semistructured gameplay videos collected on mobile devices reveal specific regions of gaze fixation as well as visual scanning patterns that differ between individuals with ASD and NT children during social gameplay. With further research and development, our system can be deployed as a diagnostic tool in diverse settings on a large scale.

## Methods

### Data Collection

**Figure 2:**
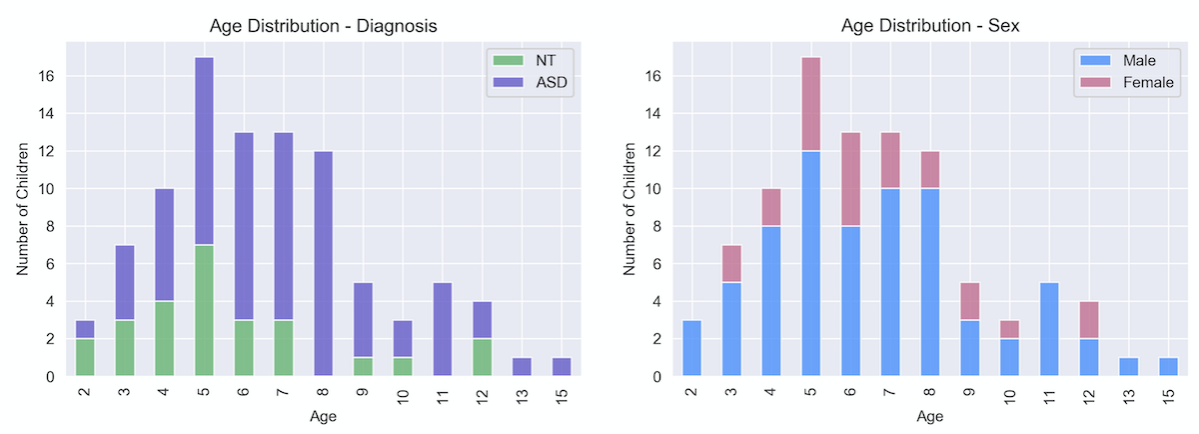
Data set information. These graphs show the breakdown of our data set by age, diagnosis, and sex. In our data set, 1 NT male failed to provide his age, and this information has been excluded from this figure. ASD: autism spectrum disorder; NT: neurotypical.

We used the GuessWhat mobile app to collect videos of children engaged in gameplay with a parent. Participants were recruited using social media advertisements and research email lists maintained by the study team. Approximately 1000 individuals proceeded to download the GuessWhat app, and we collected 449 videos from 95 children for this study. The participants ranged in age from 2 to 15 years and included 68 children (15 females, 53 males) diagnosed with ASD as well as 27 NT children (9 females, 18 males). Each child contributed a mean of 4.7 videos (SD 7.3), resulting in a total data set size of 1,084,267 individual frames and 11.1 hours of footage, presented in Figure 2. All parents consented to share their videos with our research team and completed a survey to provide the age, sex, and diagnostic status of their children.

### Data Preprocessing

Although the semistructured format of our video data set presents numerous advantages, home videos are naturally heterogeneous in quality; this results in several challenges that must be addressed prior to computational analysis. Specifically, excessive camera movement and poor lighting conditions rendered some frames in our data set too blurry for use. Moreover, other adults or siblings would often join in gameplay, resulting in multiple faces in the frame and making identification of the participating child challenging. Another major challenge arises from the lack of fine-grained annotations and ground truth labels; although the lack of eye-tracking hardware enables natural child motions and interactions, this also results in a lack of calibration information for obtaining accurate gaze locations.

**Figure 3:**
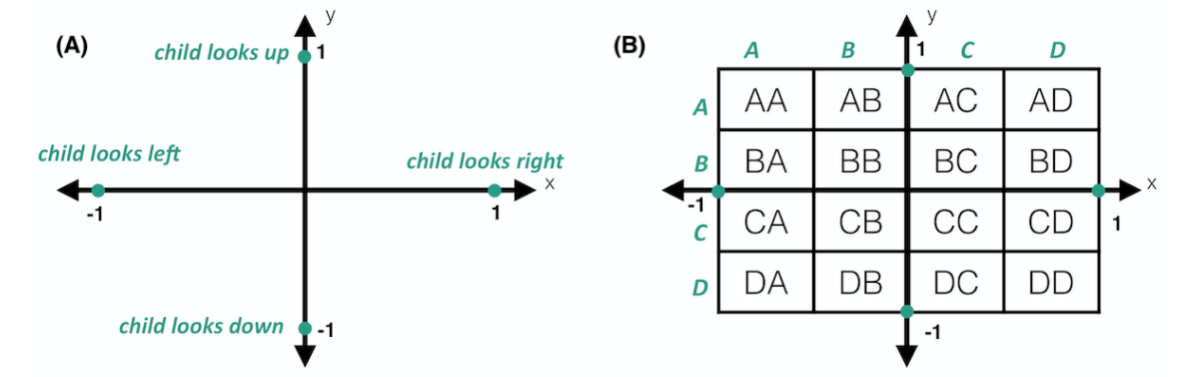
Gaze annotations. (A) Gaze coordinates range between –1 and 1 on the x- and y-axes. (B) To categorize gaze coordinates into discrete regions, we divided the gaze map into 16 buckets. Each area of interest is labeled with corresponding row and column letters.

We began our analysis with extensive quality control and data preprocessing. To preserve privacy, we annotated our data set solely using computational methods. We first used Amazon Rekognition, a powerful off-the-shelf computer vision platform developed by Amazon, to perform noisy labeling of key features in each still frame, including 30 facial landmarks and facial bounding boxes. Frames with 0 or greater than 2 faces were removed from the data set. We then used an open-source facial landmark annotation platform called OpenFace to obtain automated estimates of gaze directions [35,36]. Each frame with an identifiable face was assigned a coordinate pair (x,y) representing the direction of the individual's gaze. The value of x ranges from –1 (indicating a leftward gaze) to 1 (indicating a rightward gaze); similarly, the value of y ranges from –1 (indicating a downward gaze) to 1 (indicating an upward gaze), as shown in Figure 3. As these coordinates were assigned with respect to the smartphone camera, a frame in which an individual is gazing straight ahead into the camera is assigned a coordinate pair of (0,0)*.* If the OpenFace model demonstrated low confidence in gaze estimation values (defined as confidence below 75%) because of occluded eyes or insufficient image quality, the frame was removed from the data set; as a result, we expected the final annotations to be of high quality, but the presence of some noise and incorrect labels was to be expected. This procedure resulted in a total of 619,620 annotated frames, representing 520,536 frames from children with ASD and 99,084 frames from NT children.

Finally, to discretize gaze annotation data, we divided the coordinate map into 16 distinct areas of interest (AOIs), as shown in Figure 3. All gaze coordinates that fell within the bounds of a particular AOI were grouped together. Such an approach allowed us to identify trends in an individual's gaze fixations and scanning patterns.

### Differential Pattern Analysis

#### Gaze Fixation Patterns

Gaze fixation, which occurs when one's gaze is held on a single target for an extended period, plays an important role in social interaction by signaling communicative intent and enabling interpersonal relationships. In a dyadic social interaction, individuals usually fixate their gaze on the target's eyes. However, individuals with ASD often face difficulty with maintaining eye contact and instead tend to focus their visual attention on other regions of the target's face. Several studies involving eye trackers and visual stimuli have shown that children with ASD tend to fixate on the mouth or other body parts; this has even been observed in children aged as young as 2 to 6 months who were later diagnosed with ASD [37-39]. Eye contact avoidance, which is explicitly examined in standard clinical diagnostic examinations, can result in decreased facial identification and social engagement.

To determine the gaze fixation patterns of individuals during a single 90-second game, we used the coarse gaze annotations obtained from our preprocessed data set. For each video in our data set, we computed the percentage of time that the child fixated his or her gaze on each of the 16 predefined AOIs. A 2-sided permutation test was used at every AOI to identify statistically significant differences between the ASD and NT populations, with the null hypothesis that the fixation times for both populations followed an equivalent distribution; we calculated the difference in the mean fixation times for 100,000 rearrangements of the 2 groups. Bonferroni correction was applied to account for multiple hypothesis tests. It is important to note that because the AOIs are correlated, the Bonferroni correction is extremely stringent and will reduce the likelihood of Type 1 errors.

#### Visual Scanning Patterns

Humans tend to transition their gaze between various objects in their environments when encountering visual stimuli, a phenomenon called visual scanning. The patterns and frequencies with which humans scan their surroundings can provide insight into how individuals process the world around them. In the context of social interaction, prior research has shown that individuals with ASD vary in the way that they scan a target's facial landmarks during a social scenario, which may contribute to difficulty with interpreting emotional or nonverbal cues. This was shown by Pelphrey et al, who demonstrated that when presented with images of faces, NT individuals typically transitioned their gaze between core features, such as the eyes and nose, whereas individuals with ASD appeared to scan nonfeature areas of the face, such as the forehead and cheeks [40]. A similar study conducted by Chawarska and Shik on toddlers corroborated these findings, providing evidence of atypical scanning patterns in children with ASD when compared to their age-matched NT peers [41]. Understanding these patterns can reveal differences in the way that individuals with ASD process visual stimuli and interact in social situations.

**Figure 4:**
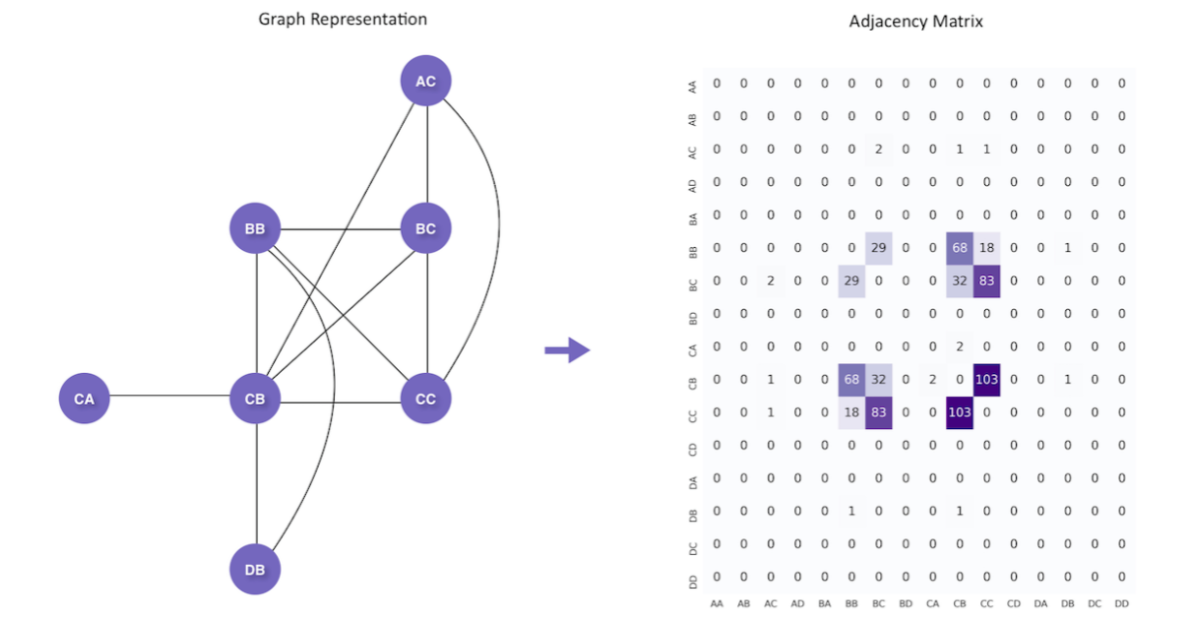
Graph model of gaze transitions. We modeled the gaze transitions in each gameplay video as a graph, which was then used to generate a 16 × 16 adjacency matrix.

Modeling gaze transition patterns as a graph problem can provide insight into the regions that children focus on while scanning their environments [42]. For each 90-second video of gameplay, we constructed a network consisting of 16 nodes *n_AA_, n_AB_, …, n_DC_, n_DD_*, with each node representing a predefined AOI. When a child shifts his or her gaze between locations on the 16-AOI gaze map, an undirected edge e=(n_i_, n_j_) is drawn between the 2 corresponding nodes. Edges are weighted by the number of transitions that occur during the game. The graph can then be converted to a 16 × 16 adjacency matrix, as depicted in Figure 4.

We computed adjacency matrices for all gameplay videos and normalized each matrix by dividing each entry by the total number of transitions. Then, we computed the average of all matrices associated with the NT individuals in our data set, resulting in a single 16 **×** 16 matrix depicting the mean percentage of transitions occurring between each pair of AOIs in a single game. This process was repeated for the gameplay videos associated with the ASD cohort. We conducted 2-sided permutation tests at each location in the transition matrix to determine if there were significant differences in the transition types between the 2 groups.

### Deep Learning Model

Next, we used deep learning techniques to measure the predictive power of gaze fixation patterns. We began by converting fixation data points into feature matrices that could serve as the input to our classifiers. We first extracted the sequence of gaze coordinates from each video using the coarse annotation procedure described in the previous section. This resulted in a vector of n ordered pairs (x,y) for every video, where n represents the number of valid frames in the video, and x and y are the gaze fixation coordinates ranging from –1 to 1. We then matched each ordered pair with its associated AOI, as demonstrated in Figure 3. This yielded a vector of *n* AOIs, representing the regions of the gaze map that each individual fixated on during a game. Next, each of the 16 predefined AOIs was assigned a number from 0 to 15 in alphabetical order, with 0 representing AA and 15 representing DD; this formed a vector of *n* integers, which we will refer to as *v*.

**Figure 5:**
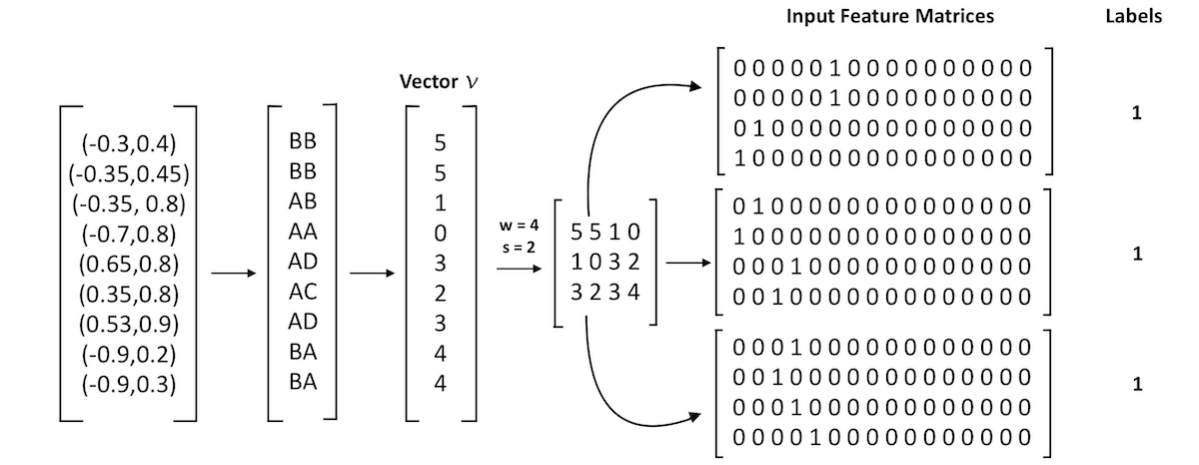
Gaze fixation feature representation. In this demonstrative example, we begin with a video consisting of 9 frames. Gaze coordinates are matched with corresponding area of interest (AOI) regions. Using a window of 4 and a shift value of 2 divides vector v into 3 feature vectors. Each feature vector is then one-hot encoded. All input feature matrices are assigned the same label.

We used a sliding window approach to divide *v* into separate vectors using 2 predefined parameters, namely window and shift. The window parameter *w* represents the number of frames included in a single feature vector; in our experiments, this value ranged from 50 to 500 frames, which roughly corresponds to 2 to 20 seconds of video content. The shift parameter *s* defines the number of elements by which the window slides between feature vectors, and we experimented with shift values between 10 and 100. These parameters allowed us to extract feature vectors from *v* consisting of *w* elements, with vectors separated by exactly *s* frames; note that if *s<w*, vectors will contain overlapping elements. Finally, we converted each *w* vector into a *w* × 16 feature matrix, with each AOI integer encoded by a one-hot vector. A demonstrative example is shown in Figure 5.

We then used deep learning models to determine if gaze fixation patterns could be predictive of ASD. We assigned 324 videos (275 ASD, 49 NT) in our data set to the training set, 71 videos (62 ASD, 9 NT) to the validation set, and 54 videos (43 ASD, 11 NT) to the held-out test set, ensuring that all videos corresponding to a single child were assigned to the same set. Input feature matrices were constructed using the approach described above. A binary label *l* ∈ {0,1} was assigned to each matrix to represent the diagnosis of the child in the associated video, with 1 representing the presence of ASD.

**Figure 6:**
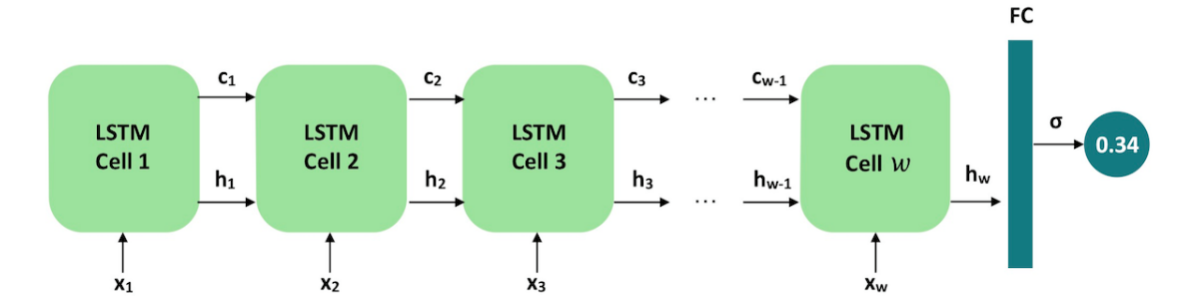
Model architecture. The model consists of a long short-term memory network with w cells. Each cell accepts a one-hot vector of size 16, represented in the figure by x_i_, and outputs a cell state c_i_ and a hidden state h_i_. The final cell is connected to a fully connected layer, which generates a single class output. FC: fully connected layer; LSTM: long short-term memory.

To exploit the temporal nature of our data set, we used long short-term memory (LSTM) networks, which are a type of recurrent neural network that can model long-term dependencies. A *w* × 16 feature matrix served as the input to an LSTM model with *w* cells; each cell accepted a one-hot encoded 16-feature vector as the input. We used the Adam optimizer with a learning rate of 0.001, a batch size of 5, and a weighted binary cross-entropy loss function. The last cell of the LSTM network was connected to a fully connected layer with a single class output followed by a sigmoid nonlinearity; this resulted in a final value ranging between 0 and 1. This value was rounded to the closest integer to determine the final prediction, as observed in Figure 6.

Finally, to characterize model performance, we report four metrics: macroaveraged recall, macroaveraged precision, weighted-average recall, and weighted-average precision. As our data set exhibits class imbalance with cases outnumbering controls, these metrics provide the most accurate representation of model performance. Macroaveraged statistics compute the arithmetic mean of performance on each class, whereas weighted-average statistics compute the weighted mean. We performed all parameter experiments on our validation set and evaluated our final best-performing models on the held-out test set.

### Ethics Approval

This study was approved by the Stanford Institutional Review Board (eProtocol number: 39562).

## Results

### Gaze Fixation Patterns Differ Between ASD and NT

**Figure 7:**
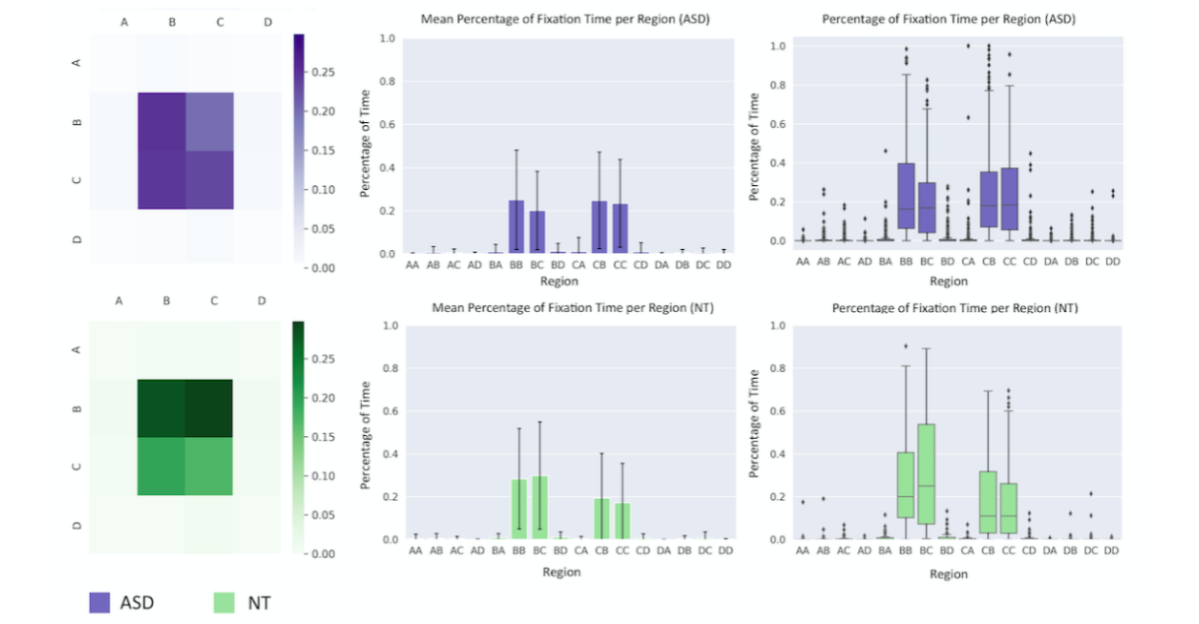
Gaze fixation results. The heat maps located at the upper left and lower left show the mean percentage of time that an individual fixated his or her gaze on each area of interest (AOI). The bar charts and the box and whisker plots show the distribution of fixation times across all videos. ASD: autism spectrum disorder; NT: neurotypical.

We first analyzed gaze fixation patterns to determine if regions of focus differ between children with ASD and NT children during a single 90-second game. Coarse gaze annotations, which were obtained using the automated labeling procedure described in the Methods section, were grouped into 16 AOIs, and the percentage of time that the child fixated on each region was computed. Figure 7 shows the mean percentage of time that the ASD and NT cohorts fixated on each AOI during a game. As shown by the heat maps, children mostly fixated on the 4 central locations BB, BC, CB, and CC, which are located closest to the camera of the mobile phone. The distributions show that several differences exist between the 2 populations; children with ASD were most likely to fixate on locations BB and CB, whereas NT children spent much of the 90-second game focusing on locations BB and BC. We conducted a 2-sided permutation test at each AOI with 100,000 permutations of the data, setting a Bonferroni-corrected significance threshold of 0.0031 to account for the 16 hypothesis tests. A significant difference in fixation distributions between the 2 cohorts was observed at location BC (*P*<.001).

### Visual Scanning Patterns Differ Between ASD and NT

**Figure 8:**
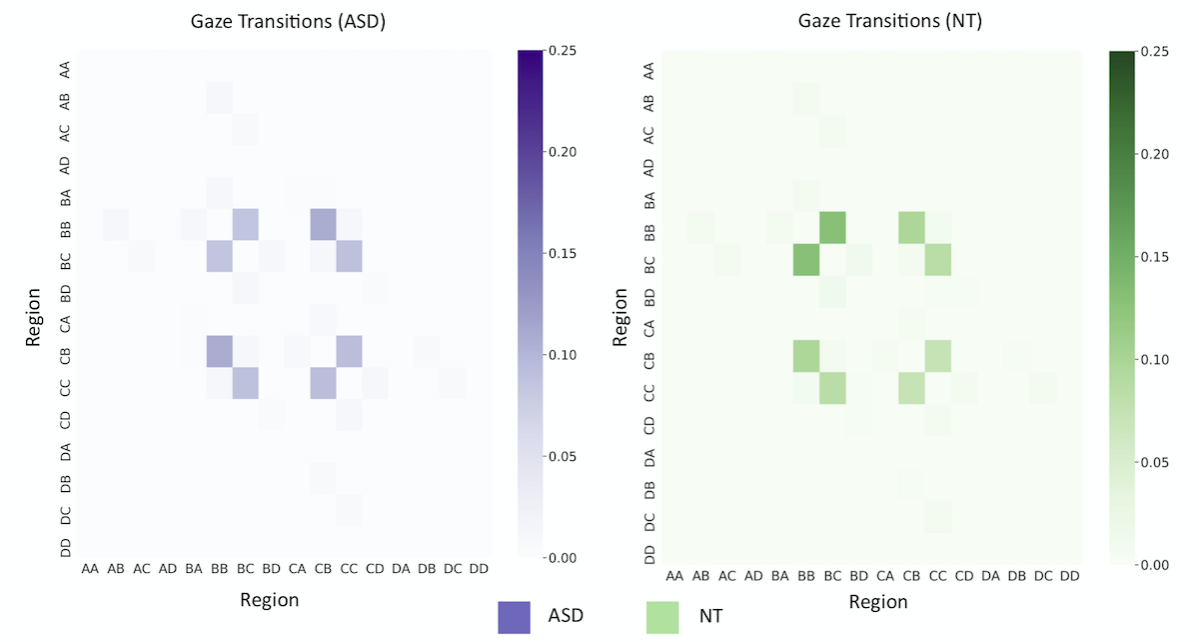
Gaze transition heat maps. These heat maps show the percentage of gaze transitions that occur between each pair of AOIs during a 90-second game. AOI: area of interest; ASD: autism spectrum disorder; NT: neurotypical.

Next, we used graph methods to analyze the ways in which participants scanned their environments during gameplay. We modeled the gaze transitions in each gameplay video as a network and computed the mean adjacency matrices for the ASD and NT populations, which are shown in Figure 8; a cell of the matrix in row *i* and column *j* represents the mean percentage of gaze transitions in a single 90-second game that occur between AOI *i* and AOI *j.* We conducted permutation tests with 100,000 permutations at each of the 61 nonzero, unique locations in the adjacency matrices; as the matrix is symmetric, the distributions for each distinct transition pair were tested for significance exactly once. We then used a Bonferroni-corrected significance threshold of 0.0008 to account for 61 hypothesis tests. Our results show that a significant difference exists in the percentage of gaze transitions between regions BB and BC (*P*<.001). As shown by the heat maps in Figure 8, 9.4% of the gaze transitions made by an individual with ASD occur between BB and BC; however, for NT children, 13% of the gaze transitions made during a 90-second game occur between BB and BC.

### Gaze Fixation Patterns Provide Mild Predictive Power

We measured the classification performance of models trained on gaze fixation patterns. Gaze fixation coordinates were encoded as one-hot vectors and passed as input to an LSTM network, which generated a single class output representing the likelihood of ASD. LSTM models were trained with a range of window and shift parameter values and evaluated on the validation set. Our results from the validation set allowed us to identify our top 3 models, which were trained with parameters (1) *w*=100, *s*=10; (2) *w*=200, *s*=10; and (3) *w*=500, *s*=10. These networks were then evaluated on the held-out test set. In Table 1, we provide precision and recall values for an LSTM model trained with those values of the window *w* and shift *s* that achieved the best performance on the validation set.

**Table 1:** Classifier performance on held-out test set with gaze fixation features.

Window (*w*)	Shift (*s*)	Macroaveraged recall	Macroaveraged precision	Weighted-average recall	Weighted-average precision
100	10	0.598	0.595	0.656	0.661
200	10	0.561	0.577	0.662	0.635
500	10	0.576	0.577	0.625	0.624

The model with parameters *w*=100 and *s*=10 demonstrated the best performance. Macroaveraged statistics are lower than weighted-average statistics, suggesting that the accuracy of prediction differs between the 2 classes. In summary, the results suggest that gaze fixation patterns can provide mild predictive power.

## Discussion

In this study, we used computational techniques to analyze home videos and obtain diagnostic insights into ASD. We collected a large data set of semistructured videos featuring children engaged in gameplay with a parent, and we analyzed 2 key markers of social engagement that have been shown to differ between children with ASD and their NT peers: (1) gaze fixation and (2) visual scanning. For each marker, we identified statistically significant differences between the 2 cohorts and demonstrated that this information could be useful in identifying the presence of ASD.

Our study demonstrates the potential that mobile tools hold for quantifying visual patterns and providing insights into ASD. Despite the presence of high heterogeneity and varying quality in our data set, the automated labeling techniques and deep learning classifiers used in this work were able to extract usable signals and identify differences in gaze fixation and visual scanning patterns between the 2 cohorts. These methods also enabled us to preserve participant privacy by avoiding the use of human annotators. Our findings support prior works that have identified social and visual engagement differences between individuals with ASD and NT individuals [37-41], and we demonstrate here that these variations can be identified using mobile tools. In contrast to previous video-based diagnostic approaches, we demonstrate that diagnostic insights can be obtained without the use of manual annotation methods, eye-tracking systems, or structured environments.

This work has some limitations. First, due to the class imbalance in our data set, the predictive accuracy of ASD differs from that of the control individuals; this is reflected in Table 1, which shows variations between macroaveraged statistics and weighted-average statistics. Additional data set augmentations will be necessary to correct this issue in future. In addition, due to camera motion and variation in the location of the smartphone relative to the parent's face, the gaze fixation maps are difficult to interpret qualitatively, and AOIs cannot be definitely matched to a parent's specific facial regions.

Future directions for this work include expanding the size of the experimental population; analyzing additional motion-based features in gameplay videos, such as limb movements and coordination; performing qualitative human-centered investigations or pragmatic randomized controlled trials to evaluate clinical usability; and evaluating the real-world diagnostic capabilities of our approach across diverse environmental settings [43-47].

Overall, this study demonstrates the usefulness of game-based mobile apps and heterogeneous video data sets in aiding in the diagnosis of ASD. With further research and development, the system described in this work can ultimately serve as a low-cost and accessible diagnostic tool for a global population.

## Abbreviations

AOI: area of interest
ASD: autism spectrum disorder
LSTM: long short-term memory
NT: neurotypical
